# Mimicking subepithelial lesions in the colon: when treating erectile dysfunction leads to an erroneous endoscopic diagnosis!

**DOI:** 10.1055/a-2686-7781

**Published:** 2025-09-05

**Authors:** Elena De Cristofaro, Jérôme Rivory, Pierre Lafeuille, Corinne Peltrault-Brulet, Alexandru Lupu, Jean Grimaldi, Mathieu Pioche

**Affiliations:** 1 Gastroenterology Unit, Department of Systems Medicine, University of Rome Tor Vergata, Rome, Italy; 2 Department of Gastroenterology and Endoscopy, Hôpital Edouard Herriot, Hospices Civils de Lyon, Lyon, France; 3 Nord-Isère, CENI ambulatoire, Bourgoin-Jallieu, France

Subepithelial lesions (SELs) of the colon are increasingly identified during screening colonoscopy and may represent a wide spectrum of benign or malignant conditions, including lipomas, gastrointestinal stromal tumors, lymphomas, or cystically dilated glands. While endoscopic resection is feasible for selected SELs, accurate diagnosis and staging remain essential, as extrinsic compression can closely mimic intramural pathology 1 2 3 .

We present the case of a 78-year-old man referred for endoscopic resection of a suspected subepithelial lesion in the cecum, incidentally discovered during screening colonoscopy. A computed tomography (CT) scan was prescribed but not performed before colonoscopy. The patient had a history of arterial hypertension and prior prostatectomy. No other relevant medical conditions or surgeries were reported.

Endoscopic examination revealed a rounded bulge measuring approximately 7 cm on the cecal wall ( Fig. 1 ), with an intact overlying mucosa and no involvement of the appendiceal orifice or ileocecal valve. Endoscopic submucosal dissection was initiated to access the lesion and achieve a definitive diagnosis, although complete resection appeared difficult to attain. An adaptative traction device (ATRACT; ATRACT Device and Co., Lyon, France) was used to better expose the submucosal layer after incision and trimming. However, the procedure was aborted upon recognition of an entirely extramural origin of the mass through a small perforation. The traction device was retrieved and the resulting mucosal defect was completely closed with endoscopic clips ( Video 1 ).

**Fig. 1:**
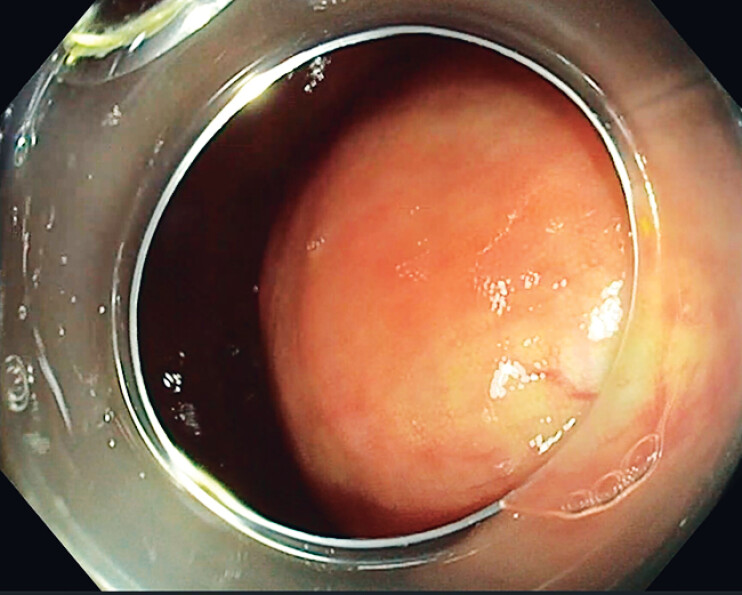
A rounded bulge on the cecal wall mimicking a subepithelial lesion.

Penile prosthesis mimicking a colonic subepithelial lesion.Video 1

A subsequent abdominal CT scan revealed the presence of an inflatable penile prosthesis, with the fluid reservoir located in the right lower quadrant, directly abutting the cecal wall ( Fig. 2 ).

**Fig. 2:**
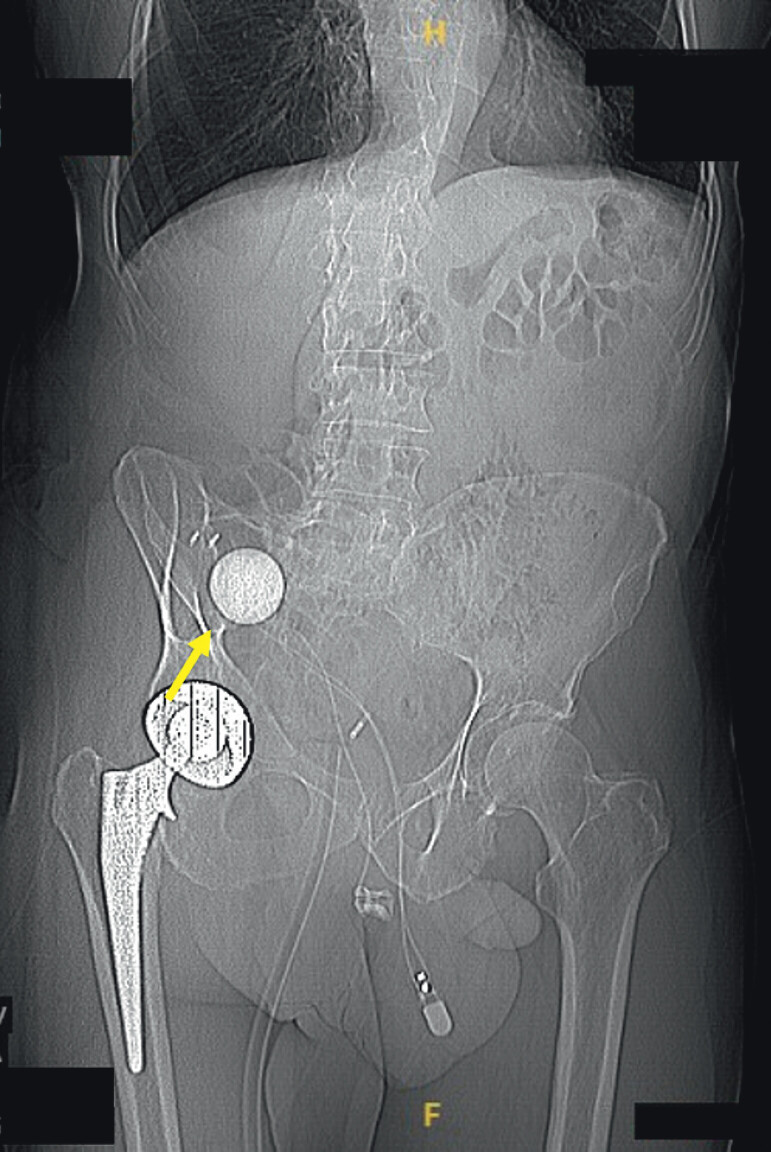
Computed tomography scan showing an inflatable penile prosthesis, with the fluid reservoir located in the right lower quadrant, directly abutting the cecal wall.

This unusual case underscores how some extraluminal medical devices can simulate true SELs during colonoscopy. In inflatable penile prostheses, the reservoir is typically placed in the lower abdomen or pelvis, often within the retropubic space. Although rare, reservoir displacement has been reported in up to 2%–3% of cases and may result in compression of adjacent organs, including the colon 4 5 .

In atypical presentations, extrinsic compression from medical devices should be considered in the differential diagnosis. Detailed history taking and cross-sectional imaging are crucial to avoid unnecessary and potentially harmful interventions.

Endoscopy_UCTN_Code_CPL_1AH_2AZ_3AZ

## Notes

Endoscopy E-Videos https://eref.thieme.de/e-videos
*E-Videos* is an open access online section of the journal *Endoscopy* , reporting on interesting cases and new techniques in gastroenterological endoscopy. All papers include a high-quality video and are published with a Creative Commons CC-BY license. Endoscopy E-Videos qualify for HINARI discounts and waivers and eligibility is automatically checked during the submission process. We grant 100% waivers to articles whose corresponding authors are based in Group A countries and 50% waivers to those who are based in Group B countries as classified by Research4Life (see: https://www.research4life.org/access/eligibility/ ). This section has its own submission website at https://mc.manuscriptcentral.com/e-videos .
